# Nurses and subordination: a historical study of mental nurses’ perceptions on administering aversion therapy for ‘sexual deviations’

**DOI:** 10.1111/nin.12044

**Published:** 2013-07-22

**Authors:** Tommy Dickinson, Matt Cook, John Playle, Christine Hallett

**Affiliations:** a University of ChesterWarrington, UK; b University of LondonLondon, UK; c University of HuddersfieldHuddersfield, UK; d The University of ManchesterManchester, UK

**Keywords:** aversion therapy, history of mental health nursing, history of sexuality, homosexuality, memory studies, nursing ethics, nursing history, oral history

## Abstract

Nurses and subordination: a historical study of mental nurses’ perceptions on administering aversion therapy for ‘sexual deviations’

This study aimed to examine the meanings that nurses attached to the ‘treatments’ administered to cure ‘sexual deviation’ (SD) in the UK, 1935–1974. In the UK, homosexuality was considered a classifiable mental illness that could be ‘cured’ until 1992. Nurses were involved in administering painful and distressing treatments. The study is based on oral history interviews with fifteen nurses who had administered treatments to cure individuals of their SD. The interviews were transcribed for historical interpretation. Some nurses believed that their role was to passively follow any orders they had been given. Other nurses limited their culpability concerning administering these treatments by adopting dehumanising and objectifying language and by focussing on administrative tasks, rather than the human beings in need of their care. Meanwhile, some nurses genuinely believed that they were acting beneficently by administering these distinctly unpleasant treatments. It is envisaged that this study might act to reiterate the need for nurses to ensure their interventions have a sound evidence base and that they constantly reflect on the moral and value base of their practice and the influence that science and societal norms can have on changing views of what is considered ‘acceptable practice’.

Male homosexuality was illegal in many countries until the mid- to late 20th century (Eisenbach 2006). In some countries, it is still illegal and individuals convicted of this ‘crime’ are subjected to harsh punishments (Ashmam 2012). In England and Wales, homosexuality was illegal from 1533 until 1967 (Dickinson et al. 2012). Furthermore, along with transvestism, it was also considered an antisocial ‘sexual deviation’ that could be ‘cured’ until 1992, when the World Health Organization finally dropped the term ‘homosexuality’ as a diagnosis with the publication of the *International Classification of Diseases Edition 10 Classification of Mental and Behavioural Disorders* (WHO 1992).

The most common treatment used in a bid to cure such individuals was aversion therapy with electrical shock or apomorphine to induce vomiting (Smith, King and Bartlett 2004; Dickinson et al. 2012). Patients who received this treatment reflected on it as ‘barbaric’; some thought they were ‘going to die’, while others recalled ‘the excruciating pain’ of the electric shocks (Dickinson et al. 2012, 1349). Although nurses were instrumental in administering these distressing and painful treatments (James 1962; Seager 1965; Johannesburg Star 1968), there is a paucity of information about this now-discredited nursing practice.

Nursing continues to struggle with the sexuality of patients (Dickinson et al. 2012). Knocker (2012) argues that issues of trust and safety are critical to older gay, lesbian, bisexual and transgendered (GLBT) people when they seek healthcare services. However, many older GLBT people are reluctant and fearful to contact healthcare facilities because of their historical poor treatment by such services (DoH 2007; Hughes 2007; Dickinson et al. 2012). Many fear that they will have to revert to being ‘in the closet’ or be on the receiving end of intolerance or bigotry if they have to access healthcare or aged-care services (Knocker 2012, 12).

An understanding of the history of GLBT people is important if nurses are to provide person-centred care or undertake life story work with these individuals (Ward, Pugh and Prince 2010). In our companion paper (Dickinson et al. 2012), we explore the experiences of male^1^ patients in the UK who underwent aversion therapy to change their sexual orientation. Here, we explore nurses' perspectives on providing these therapies to cure individuals of what were deemed to be their sexual deviations. The period that this study examines is from 1935 to 1974. The period began with the publication of the first official report on aversion therapy being utilised to treat homosexuality. The report was by Louis Max, a psychiatrist, who required a homosexual patient to fantasise about an attractive same-sex sexual stimulus in conjunction with receiving an electric shock (Max 1935). The period ends in 1974 with the seventh printing of the American Psychiatric Association (APA) *Diagnostic Statistical Manual* (DSM) version II, which removed homosexuality as a category of psychiatric disorder. Although published in the USA, this manual was widely utilised in the UK to aid healthcare practitioners to diagnose mental illness.^2^

The article aims to offer some interpretations for why nurses administered these painful and distressing treatments. Furthermore, to reiterate the need for nurses to ensure their interventions have a sound evidence base and that they constantly reflect on the moral and value base of their practice and the influence of ‘science, societal norms and contexts on changing views of what is regarded as ‘acceptable practice’ (Dickinson et al. 2012).

## BACKGROUND

While one can view these treatments as brutal, an examination of the context in which they were developed in the UK, may help explain why some nurses believed in their efficacy. In the mid-20th century, psychiatrists cared for patients for whom, in many cases, there was no effective treatment beyond custodial care (Adams 2009). With the inauguration of the 1930 Mental Treatment Act, UK psychiatry was working under a new legal framework. In keeping with the nomenclature of this new Act, the role of psychiatry was to treat and cure patients, restoring them to their homes and employment (Pressman 1998). During the same time period, the therapeutic options for treating psychiatric patients were also being transformed. A spirit of optimism emerged within psychiatry, as new and often distinctly unpleasant somatic treatments were introduced, providing hope to psychiatrists – and nurses – who previously had few effective treatments to draw on.

The four most significant somatic treatments were insulin treatment, cardiazol treatment, electroconvulsive therapy (ECT) and leucotomy (Shorter 1997; Kragh 2010). Thus, from having no physical treatment beyond sedation for patients, four treatments were available, and a wave of enthusiasm resulted in the adoption of these therapies before proper evaluation had taken place (James 1992). Nurses administered or assisted with these invasive and distressing somatic treatments, alongside psychiatrists' rudimentary justifications. Therefore, one could argue that the exposure of nurses to such treatments may have normalised the implementation of ‘therapeutic’ interventions which caused distress to patients receiving them. Some nurses may have accepted that disturbing interventions such as aversion therapy were normal, morally acceptable, and part of a larger venture that promised positive outcomes: in essence, the ends justifying the means. Furthermore, the work of nurses was also largely constrained by the asylum-type conditions in which they worked. Nurses were swamped by the influential culture of the institution, which dictated that ‘nursing was learnt by watching the example of others, based on ‘common sense’ assumptions and concerns with neatness rather than on research-based theory’ (Hopton 1999, 360).

Post–World War II, fears surrounding homosexuality in the UK acquired a particularly powerful resonance, and narratives of sexual danger as corruption predominated in public discourse (Houlbrook 2005; Dickinson et al. 2012). Treatments for sexual deviations in the UK were precipitated by pejorative political rhetoric towards homosexuals, with the Home Secretary (Sir David Maxwell Fyfe) remarking in 1952 that ‘homosexuals make a nuisance of themselves’ (Jivani 1997, 127). The media were also offering normative judgements about appropriate and inappropriate male social identities and same-sex behaviour, which included *The Sunday Pictorial* portraying homosexuals as ‘evil men’ (Sunday Pictorial 1952).

Treatments to cure homosexuality and transvestism were usually carried out in National Health Service (NHS) hospitals using two types of aversion therapy. Chemical aversion therapy involved inducing vomiting by utilising a powerful emetic, apomorphine, and electrical aversion therapy for transvestism involved the patient standing on an electric grid dressed in women's clothes and receiving electric shocks through his feet. In the case of homosexuality, pictures of nude men as the erotic stimulus were used as the subject to be averted (Dickinson et al. 2012). Although in some cases electric shocks were given automatically in response to increases in penile erection, measured by a plethysmograph (Bancroft 1974; Dickinson et al. 2012), more often these were administered by a nurse, psychiatrist or psychologist.

Patients who received chemical aversion therapy were treated as inpatients on psychiatric wards due to the intensive nature of the therapy and the side effects of nausea and dehydration (Smith et al. 2004). Those receiving electrical aversion therapy were treated as outpatients, in some cases for over a year (Dickinson et al. 2012). In the majority of cases, treatment efficacy was based on patient self-report, and many reported no effects in altering their sexual desires. Indeed, in some cases, these treatments had negative, long-term effects on patients (Smith et al. 2004; Dickinson et al. 2012). Nevertheless, the medical press and wider media sanguinely reported successful outcomes to these spurious interventions (Max 1935; James 1962; The Observer 1962).

## THE STUDY

### Aims of the study

The aim of the study was to examine the experiences of nurses administering ‘treatments’ to change sexual deviation in the UK. It looked in detail at nurses' perspectives on providing these treatments.

### Design

For a fuller description of the research methods used, please see our companion paper Dickinson et al. (2012). This was a UK-based study based on oral history interviews. The main research method used was oral history, which can be defined as ‘a systematic collection, arrangement, preservation and publication…of recorded verbatim accounts and opinions of people who were witness to or participants in events’ (Moss 1974, 7). Face-to-face oral history interviews were conducted with participants; these were audio-taped and transcribed for historical interpretation.

### Sample

Purposeful sampling was used to select participants for the study (Boschma et al. 2007). Inclusion criteria were that participants were qualified nurses who had administered medical treatments in a bid to cure individuals of their sexual deviations, as well as the ability and willingness to communicate this experience. Fifteen mental nurses^3^ (eight men and seven women) aged between 63 years and 98 years were interviewed. Two commenced nursing in the 1930s, five in the 1950s and eight in the 1960s. All the nurses had worked in NHS hospitals and had administered aversion therapy on acute inpatient admission wards or outpatient clinics between 1959 and 1974. Eleven were involved in administering chemical aversion therapy, three had administered electrical aversion therapy and one had administered both. All of the nurses identified themselves as having Caucasian ethnicity. One was originally from France, and three were originally from Ireland; the rest were from the UK. Seven participants were recruited from an article published in *Mental Health Practice* (Dickinson 2010) with others recruited by means of snowball sampling.

### Data collection

Data were collected between December 2009 and December 2010. Perks and Thomson (2000, 7) argue that the core of oral histories is the interview itself and that to interview successfully requires skill. They argue that interviewers must possess the capacity to show understanding and sympathy for the interviewees' point of view, ‘and above all, a willingness to sit quietly and listen’. Thompson (2000) posits that there are two approaches to interviewing: first, the ‘objective/comparative’, usually based on a questionnaire or a highly structured interview; and second, a free-flowing dialogue between interviewer and respondent, with no set pattern, in which conversation is followed wherever it leads. To strike the balance between these two approaches, face-to-face semi-structured interviews were conducted by the principle investigator (PI), which lasted a maximum of two hours.

### Ethical considerations

Ethical approval was obtained through the University Research Ethics Committee. Key ethical issues related to confidentiality and anonymity of the participants and ensuring informed consent. All participants were given a participant information sheet and had the study fully explained to them. They were given the opportunity to ask any questions, and if they still wanted to participate, they signed a consent form. One condition of ethical approval was that the PI had to ensure a counsellor was available for participants should they become distressed during the interview; however, all participants declined this when offered to them (Dickinson et al. 2012).

### Data analysis/historical interpretation

Prebble (2007, 23) argues that in order to begin analysing oral history interviews, one must engage in ‘a process of immersion, questioning, contrasting and comparing’, which requires openness and humility. At times, the interpretive process started before the PI had even met the participants face-to-face. Potential interviewees were contacted over the phone to arrange a convenient time and place to conduct the interview (all chose to be interviewed in their own homes), and during this dialogue, the PI and the interviewees often became engaged in conversation about the topic in question. This provided the PI with an opportunity to reflect on and analyse these conversations before they actually met, allowing him to follow up on any questions that had emerged from the initial telephone conversation during the actual interview. The interviews were audio-tape recorded and transcribed for ease of analysis. Green (2004, 11) argues that the way we tell stories, and the language we use, is not always as straightforward as it might first appear. It is ‘rarely a transparent or neutral medium’. Therefore, the interviewees' emotional responses, body language and their levels of engagement during the interview were also noted.

The interpretive process continued as the PI listened to the audiotapes to transcribe the interviews. He repeatedly visited and revisited the transcripts and, as new questions and themes emerged from his analysis, he often relistened to the interviews. As he became more absorbed in the details, reflections and experiences of both the nurses and former patients, he discovered that their testimonies offered unanticipated and important understandings of their shared culture and identity. In parallel to other oral history studies, the participants' testimonies sent him back to the written primary sources to clarify dates, official views or political and social contexts (Prebble 2007). Occasionally, the PI was directed back to the interviews to establish how events or practices appeared to the participants as he discovered new data in the archives.

Researchers must always be concerned about the potential emotional effect that alternative readings and interpretations of personal testimonies may have on the living participant (Borland 1998; Dickinson et al. 2012). As discussed in our companion paper (Dickinson et al. 2012) to work around this and to ensure rigour in the interpretation phase, it was important to work in alliance with participants throughout both the data collection and interpretation phases. In the light of this, where possible, participants were recontacted by phone or e-mail for more information or to clarify their perceptions of certain matters or events. However, this was not always possible with all participants as some sadly died in the period between data collection and interpretation.

## RESULTS

Fifteen mental nurses who met inclusion criteria were interviewed, aged between 63 and 98 years.

### Following orders and subordination

The majority of nurses in this study identified that their role was to carry out the orders of medical staff or their nursing superiors uncritically and without question:

Our job to all intents and purposes was to follow the doctors' order… [Pause]…I mean you have to understand the power the doctors, Nursing Officers and Matron had in those days. You stood up to attention, for example, when the doctor came on the ward. Likewise, when the Matron or Nursing Officer came on your ward, they were checking that all beds were in line, with the wheels pointing in exactly the same direction….erm…the beds, well they had to be turned down from there to there [shows distance with hands] exactly– they even measured to make sure it was. No one ever told me why we had to do that. I don't suppose anyone ever thought to ask. It was the same with aversion therapy, I didn't ask why – I just did it. It was the doctor who needed to know the why's, what if's and maybe's in my day.

[1]

Some nurses in this study felt completely unskilled to nurse the homosexuals or transvestites when they were admitted to their wards:

I didn't really understand what we were doing, none of us nurses did. We knew we were trying to get him to go for women instead of the men, but that was about it. The doctor brought the young man in and told us what we were going to do. I didn't really think anymore about it, just got on with it – it was my job. I thought the doctor knows what he is doing, so it must be in the patient's best interests. In those days you didn't really ask questions, and you just did what the doctor told you to do really. When I think about it, we did not have any real knowledge to base this practice on, other than it was very experimental, not like you have now: my granddaughter is a nursing student and is trained to “question practice” [laughs], even doctors! My god! You would never do that in my day, you would not have dared. They had overall superior knowledge, or at least that is what we were trained to believe, and subsequently thought. That is what they thought of themselves too; we did what they said, because they could not possibly have been wrong.

[5]

When nurses did ask questions, they were often regarded as, ‘[…] audacious and impudent’ [3]. One participant muses on the reasons for this:

I remember the first time I saw it [aversion therapy for transvestism] I thought it was barbaric. And I remember asking the Charge Nurse: “By administering the shock where is the treatment?” And of course this was regarded as insolent and impertinent question at the time. Because it went outside the training and the training was set pieces of knowledge you regurgitated in exams, and if you were able to do that you were a competent nurse and not awkward. So it was in fact an education and training in avoiding awkwardness, because that is how you ran a very stable institution. So I just got on with it. I think the nurses and patients blinded themselves to the doctors' treatment.

[13]

### Task-orientated practice and avoiding responsibility

The terminology that some of the participants used could suggest that they were practicing in a very task-orientated manner:

Nursing was very regimented and task orientated in those days – not least the care of patients receiving aversion therapy – particularly those receiving chemical aversion therapy. We seemed to have it pretty boxed off, and took in turns to either do and have responsibility for is [sic] obs or give the injections.

[1]

Meanwhile, some nurses avoided responsibility for such patients by distributing nursing interventions to others involved in the patients care:

[…] cause [sic] it was dammed hard work looking after those homosexuals, you were on the go all night, you had to keep on at this bloke to keep taking this that and the other – observations – I mean blood pressure and testing his water, you know that went round the clock. I didn't give him the injections, we shared the jobs, my colleague gave the injections and I took his observations.

[14]

### Antipathy vs. empathy

It appears that the nurses' role in aversion therapy was to make the treatment as unpleasant as possible and not to build up strong therapeutic relationships with these patients:

We didn't have to talk to ‘em [sic]. If he was emotionally distressed it still went on. As long as his body was alright…I mean as long as you were shaking ‘em [sic] up you know? Well, you were doing the work. The work's being done if he was shook up. I suppose we were being cruel to be kind. This is what we believed was the best cure for them. We thought we were doing good.

[7]

Another participant concurs and recalls the lack of empathy this patient group received:

I don't ever recall any meetings or ward rounds to discuss these [homosexual and transvestite] patients. There was a distinct lack of empathy and sensitivity to this patient group. They were seen as trouble makers and deviants, who were put on this earth to annoy and cause trouble for everyone around them. There was a belief that they were fully responsible for entering into the culture in which they drifted.

[9]

Nevertheless, one participant recalled, ‘I had always prided myself for showing the utmost of respect, courtesy and empathy for the patients in my care’. [4]. Meanwhile, another commented ‘Even though we were not really supposed to, I tried to sit down with the patient and offer them support’ [13].

### Nurse education

Some of the nurses in the study recalled the education they received regarding homosexuality and transvestism, and its limitations in regard to equipping them with the skills required to actually nurse these patients. One participant recalls, ‘They were very good at describing sexual deviants, but not so good at giving us the skills to actually nurse these patients’ [12]. Other nurses recalled their education regarding sexual deviants:

In lectures the tutors would lump abnormal sexuality into a common pot, so the fact that you might have paedophile tendencies, or you might be gay, was all the same, it was all deemed to be wrong. They would be lumped into this bag of, you know, deviants if you like.

[11]

I do remember a lecture that was given at the ******** [name of the hospital]. This lecture was on deviancy, and as part of deviancy, homosexuality and transvestism came up. It was talked about in the same vein as criminality. Homosexuality and transvestism were included in a bunch of lectures that were given by a consultant. Now how it was presented to us was that these behaviours were deviancies, and they came as part of a package of deviancies. They were seen as a denial of who you were, an adoption of a lifestyle that you chose, rather than had to. There was also gain to be had from behaving and acting as a homosexual or a transvestite, but they were not normal – that was the point that was trying to be got across.

[13]

## DISCUSSION

### Dissonance between reality and rhetoric

The participant's gender did not appear to make any difference to their perspective on providing these treatments. The results appear to suggest, however, that the majority of the nurses in this study believed that their role was to subordinately follow any orders they had been given from higher authority. However, an article published in the *Nursing Times* in 1965 entitled ‘Aversion Therapy in Psychiatry’ suggests that there was evidence of an immense gulf between the prescriptions of theory, the intentions of policy and the realities of practice. A quote from this article urges nurses not to merely accept doctors' orders, but make the decision to partake in this aspect of their clinical practice only after they have reflected on their own values regarding it:

If a nurse is asked to participate in this type of treatment it is most important that she considers her view on the matter rather than merely accepting orders. One must consider one's own motives when applying this treatment. There may be conscious or unconscious reasons for wishing to inflict pain, either on people in general or on a particular group, such as homosexuals in particular. […] In its present stages the treatment is experimental, and until it has been found either to fulfil its purpose or, on the other hand, to be unsuccessful, it must remain a necessity for all concerned with its administration to look at it carefully and make their own decisions about their participation.

(Seager 1965)

The way nurses worked on the wards appeared to rest on the preference of the supervising doctors, sister or charge nurse. They were often kept unaware about the patients and the reasons they had been admitted. Case notes were kept off the wards in the central office, and only doctors had access to them (Nolan 1989). The vast majority of nurses were not party to the wider debates about treatments, which were taking place outside the mental hospitals. Within the hospitals, rarely did nurses participate in case conferences, discuss patients' treatments or diagnoses, or assess the progress of patients (Nolan 1993). The culture of many mental hospitals – and hence nursing – was custodial, ritualised and impersonal. Nurses were expected to provide interventions with little, if any, consideration of the efficacy or theoretical underpinning of these. Staff discipline was inconsiderately managed with nurses obeying their superiors' orders to avoid being publically humiliated in front of colleagues and patients (Beardshaw 1981).

### Nurses and obedience: a comparison with nurses in Nazi Germany

Nurses' involvement in aversion therapy is not the only example of their adoption of unethical practices and behaviours in subordination to higher authority. This justification has been used as a shield by nurses in supporting their unethical practices in a number of historical contexts not least in Nazi Germany (McFarland-Icke 1999; Berghs, Dierckx de Casterle and Gastmans 2007). It is of course crucial to emphasise the different context and that none of the nurses in this study knowingly murdered patients in their care as nurses under Nazi rule did.^4^ Nevertheless, both sets of nurses administered what could now be deemed to be brutal treatments. Nurses in this study commented that they merely followed the orders of higher authority. Therefore, given that many nurses under Nazi rule offered the same reasons for their behaviour, there could be something to learn from a comparison.

Under Nazi rule, nurses were involved in various stages of euthanasia programmes (McFarland-Icke 1999; Berghs et al. 2007). In the children's euthanasia programmes, nurses actively abetted in executing young people through injections of morphine and scopolamine, by starvation, or by overdoses of other medications (Benedict and Georges 2006; Berghs et al. 2007; O'Donnell 2009). Nurses selected and helped eliminate concentration camp prisoners in the later Operation 14 f 13; they also participated in the implementation of the ‘Final Solution’ and in mass sterilisation programmes (Biley 2002; Berghs et al. 2007). They assisted with compulsory medical experiments on people, refused to admit and treat Jewish and homosexual people, and were, overall, ‘involved in all phases of the systematic annihilation of masses of people’ (Biley 2002, 1567).

This brief depiction of some of the events of the Holocaust cannot do justice to the extent of suffering that was experienced. However, it does begin to illustrate some similarities with the nurses and patients in this study. Indeed, one patient who received treatment for homosexuality used this parallel as a metaphor when he reflected on his treatment as ‘a barbaric torture scene by the Gestapo in Nazi Germany to extract information from me’ (Dickinson 2012, 15). It is not surprising that the participant made this connection. Pattinson (2007) states that among other torturous interventions, the Gestapo deprived prisoners of sleep, subjected them to electric shocks, denied them light, food and medical treatment and kept them in solitary confinement. The treatment of sexual deviations with aversion therapy used a combination of all of the above (Max 1935; James 1962; Oswald 1962).

### Limiting culpability

To make the administration of brutal treatments tolerable, the role of morality had to be limited, and in some cases, this was carried out by distributing the individual accountability that nurses could take for their actions. The terminologies used by some participants in this study – that is, ‘those homosexuals’, ‘dammed hard work’, ‘the sexual deviant’, ‘sort them out’ and ‘It must have been awful for the other patients’ – could suggest two things: first, that nurses were practicing in a very task-orientated manner, and second, that as with the nurses in Nazi Germany, they limited their integrity using dehumanising and objectifying language and focused on administrative tasks, rather than the human beings in need of their care (McFarland-Icke 1999; Berghs et al. 2007). Meanwhile, other nurses in this study discussed the distribution of specific tasks involved in nursing these individuals as a means of avoiding participation.

The Nazi euthanasia projects had to be a secretive collective endeavour, with each individual nurse following orders and doing a very specialised administration or technical task (Berghs et al. 2007). Berghs et al. (2007, 846) go on to argue that the adoption of very specialised tasks enabled nurses to focus on task performance, measuring their nursing responsibilities in ‘narrow terms of efficiency, productivity or competence’. McKie (2004) proposes that focussing on the detached nature of a task allows an emphasis to be shifted from victims (patients) to perpetrators (nurses) with the focus on the difficulties inherent in responsibilities for tasks rather than patients.

Nevertheless, Steppe (1997) argues that some Nazi nurses appeared to salve their conscience in relation to administering lethal injections to patients by treating them with tenderness and respect as they gave the poison. Moreover, some nurses in this study also appeared to salve their morality in regard to administering aversion therapy by ensuring they displayed empathy and compassion to their patients who were receiving it. This could offer an explanation for some nurses' acceptance and participation in aversion therapy.

### Antipathy: a beneficent intervention?

The crux of a mental health nurse's role is to display unconditional positive regard and empathy to the patients in their care (Dickinson and Hurley 2012), and Hunter (1956, 99) highlights this as the very function ‘to which mental nursing owes its inception – that is, to counter alienation by sustained, kindly human understanding and contact’. However, some nurses in this study were not displaying empathy to the patients in their care; indeed, one could argue that they were displaying the opposite – antipathy. Indeed it was believed that aversion therapy was more effective if the patient experienced a ‘maximum emotional crisis’ (Oswald 1962, 198).

Some nurses in this study genuinely believed that aversion therapy, and the antipathetic behaviour that accompanied it, was the most therapeutic intervention for these individuals. In essence, their motivations to participate in such treatments appear to be embedded in a notion of beneficence. However, one could argue that even if their practice was based on a notion of beneficence, they were not upholding the principle of nonmaleficence; the treatments were very traumatic and painful for the patients receiving them. Therefore, such notions of beneficence may have their roots in paternalism, believing that nurses knew what was best for their patient (Rumbold 1999). Gillon (1986) couches this notion as ‘beneficent paternalism’: when healthcare providers tell patients what is good for them without regard to the patient's own needs or interests.

### Educating mental nurses regarding ‘sexual deviations’

There is a dearth of literature in nursing textbooks during this period, which discuss sexual deviations. The texts that do discuss homosexuality and transvestism do so under the auspices of ‘Sexual Perversions’, ‘Sexual Anomalies’ or ‘Sexual Disorders’ (Bachelor 1962; Ackner 1964). The emphasis in these texts appears to be on describing these disorders, rather than educating nurses how to actually care for this patient group. Furthermore, the testimonies from the participants in this study highlight that the education nurses received regarding homosexuality and transvestism had a clear emphasis on viewing these people as abnormal, with little importance paid to equipping nurses with the skills required to care for these individuals. Indeed, one participant recalled, ‘I remember my colleagues and I being totally unprepared for dealing with and talking to them [homosexuals and transvestites] when they arrived on the ward’ [5]. This was further compounded by the wider debate regarding how to view the sexual deviant that was being pressed by the media and literary works (Rees and Usill 1955; The Scotsman 1959). Nurses were not receiving an education that presented a coherent and robust knowledge regarding these individuals.

### Psychological insights into the nurses' actions

Goldhagen (1996) proposes that in some instances, obedience to higher authority is pursued due to an individual's self-interest, which is conceptualised as career advancement or personal enrichment at times in total disregard of other considerations. However, it could be argued that this explanation is untenable for the majority of nurses in this study – not least the state enrolled nurses^5^ (SENs) and those who remained staff nurses for their whole careers. These nurses had no organisational or career interests to advance by their involvement in aversion therapy. They were not striving for promotion. Therefore, this ‘self-interest’ argument fails to accord with the majority of nurses' testimonies in this study.

Milgram (1974) argues that most humans in general are blindly obedient to authority, in some cases reflexively obeying any order, regardless of its content or consequences. However, Kelman and Hamilton (1989) argue that this interpretation is indefensible as all obedience depends upon the existence of a favourable social and political context in which individuals deem the commands that have been issued not to be a gross transgression of their intrinsic values and their central morality. Indeed, Goldhagen (1996) suggests that if favourable social and political contexts are not in place, people will seek ways, granted with differential success, not to violate their deepest moral beliefs and not to undertake such grievous acts.

When one revisits the pejorative political rhetoric and media headlines that were in abundance during the period under study, it could be argued that these were broadly in favour of aversion therapy to treat sexual deviations, providing a favourable social and political context to these treatments. This could corroborate the influential impact that the media and political rhetoric had on the nurses' morality in relation to their participation in such therapy and provides a further context to, at least in part, explain their subservient behaviour. As one participant remarked, *‘*I remember the press discussing “how a doctor had cured a homosexual”…I suppose the fact it was printed for all to see was confirmation of the good work we were doing.’ [5].

## LIMITATIONS

The participants in this admittedly small-scale study may not be representative of all those who administered treatments for sexual deviations. Some individuals may have been reluctant to take part, or may have died or emigrated. Further, while no nurses in this study reported that they steadfastly refused to participate in these treatments, in reality, there may have been nurses who did. Meanwhile, some nurses may have had sinister – yet unrevealed – motivations underpinning their participation in aversion therapy. Finally, the sexual orientation of the nursing participants was not elicited, and the lack of this information could be a potential limitation. In the light of this, the PI is currently undertaking further research in this area. Therefore, this study cannot address the full reality of the meanings that all nurses attached to these treatments.

## CONCLUSION

This article contributes to a relatively new body of historical literature regarding the work and practice of mental health nurses in the UK. In doing so, it adds fresh material and a new perspective to the documented history of mental nurses' experiences and perceptions of the ‘management’ of individuals belonging to stigmatized groups. It is envisioned that this study can offer insights into the way nurses may behave when a particular set of social, political and contextual factors are at play. First, the culture of many mental hospitals – and their nurses – was custodial, impersonal and ritualised. The work of nurses was also largely constrained by the asylum-type conditions in which they worked, and the character and quality of patient care was largely influenced by the dominance of medical staff that had power over the institution and the practices of nurses within it. In addition, due to their limited knowledge base, some nurses believed that it was pertinent for the welfare of a patient that nurses simply obey orders. They took on the status offered to them of an obedient follower of orders.

Furthermore, nurses as others in the general public were exposed to prejudicial attitudes towards homosexuals and transvestites being expressed through the media, literary, medical, sociological and legal discourses. The derogatory rhetoric regarding sexual deviants during the 1950s and 1960s may have created a favourable social and political context for these treatments as well as reinforcing notions of beneficence, which underpinned the practice and participation of nurses. Without judgement, this resulted in a set of actions, which, on reflection, were brutal and harmful to the patients receiving them and both evidentially and ethically unjustified. One could argue that what was lacking at the time was a culture in which nurses possessed the knowledge base and self-esteem to voice their concerns and question those in higher authority.

This study is timely as reports of disturbing allegations of nurses participating in electrocutions, whippings, operations without anaesthetics and other brutal treatment of patients in Syrian Military Hospitals are published (Los Angeles Times 2012; The Daily Mail 2012). Furthermore, in May 2012, history was made when Ms. Lesley Pilkington, a psychotherapist, was found guilty of malpractice after trying to ‘cure’ a homosexual patient in her care (The Independent 2012). Therefore, it is envisaged that this study might act to reiterate the need for nurses to ensure their interventions have a sound evidence base; that they constantly reflect on any orders they may have been issued with and the moral and value base of their practice; and the influence that science, societal norms and contexts can have on changing views of what is considered ‘acceptable practice’. We can learn much from studying aspects of our profession's past in which our actions, even if countenanced by the context in which they were situated, did not serve patients and society well.

Those who care for older GLBT patients need to be mindful of some of the struggles this minority group may have lived through, ensuring that they are nonjudgmental and accepting of their patients' sexual orientations and current genders. Finally, it is anticipated that this study may enable nurses not only to review their own experience of nursing these client groups, but also to envision alternative possibilities for constructive and caring interventions for these patients in their care. It is essential that nurses develop GLBT-friendly environments that enable these individuals to disclose and express their identities (where they feel it is appropriate) enabling them to feel supported and respected.

## Footnotes

1 All former patients who participated in this study were male at the time they received the treatment. That is not to say that women were not subjected to psychiatric evaluation or advised to undergo these treatments; they were (Jivani 1997). However, of all the reported cases in the medical literature, only one published study discussed aversion therapy being administered to women (McCulloch and Feldman 1967). Furthermore, no women came forward as research participant for this study. It is important to note that while female sexual deviation – predominantly prostitution – was inscribed within forms of investigation that mirrored the regulation of male sexualities, lesbianism remained invisible in the law (Houlbrook 2005). When we consider that one of the main ways in which men were referred for these treatments was through a court order, this could offer a context to explain the limited response from females to this study and their limited presence in the literature.

2 It is important to note that the World Health Organization only decided to drop the term ‘homosexuality’ as a diagnosis in 1990. It was eventually removed from their diagnostic manual in 1992, with the introduction of the *International Classification of Diseases Edition 10 Classification of Mental and Behavioural Disorders* (ICD-10). Nevertheless, there is a paucity of literature describing treatments for homosexuality after 1974, as the publication of the seventh printing of the DSM II, combined with a fresh gay liberation movement in the 1970s, was seminal in the curtailment of these treatments. Additionally, no participants in this study reported receiving treatments after this date. Therefore, the decision was made to end the study in 1974.

3 Given that, for the majority of the period explored in this study, the most commonly utilised term was “mental nurse”; this term will be used throughout the article for consistency.

4 While it is important to note that the aim was never to murder patients who were receiving treatments for their sexual deviations, there is at least one reported case where a patient died as a result of the chemical aversion therapy he received to ‘cure’ him of his homosexuality (Smith et al. 2004, 3).

5 SENs undertook a two-year nurse training course, which enabled them to enrol as a second level nurse with the General Nursing Council in the UK. Their course was a simplified version of the longer training offered to state registered nurses.
